# Light sheet fluorescence microscopy pipelines for imaging and analysis of the human placental vasculature across gestation^†^

**DOI:** 10.1093/biolre/ioag166

**Published:** 2026-07-31

**Authors:** Mary E Spring, Isha Ramanlal, Adrienne K Scott, Toby E Jackson, Jacqueline M Ross, Alys R Clark, Anna L Boss, Joanna L James

**Affiliations:** Department of Obstetrics, Gynaecology, and Reproductive Sciences, Faculty of Medical and Health Sciences, The University of Auckland, Auckland, New Zealand; Department of Obstetrics, Gynaecology, and Reproductive Sciences, Faculty of Medical and Health Sciences, The University of Auckland, Auckland, New Zealand; Department of Aerospace and Mechanical Engineering, University of Notre Dame, Notre Dame, IN, United States; The Auckland Bioengineering Institute, The University of Auckland, Auckland, New Zealand; Biomedical Imaging Research Unit, The University of Auckland, Auckland, New Zealand; The Auckland Bioengineering Institute, The University of Auckland, Auckland, New Zealand; Department of Obstetrics, Gynaecology, and Reproductive Sciences, Faculty of Medical and Health Sciences, The University of Auckland, Auckland, New Zealand; Department of Obstetrics, Gynaecology, and Reproductive Sciences, Faculty of Medical and Health Sciences, The University of Auckland, Auckland, New Zealand

**Keywords:** placenta, light sheet fluorescence microscopy, feto-placental circulation, placental vasculature

## Abstract

Over the first trimester, vasculogenesis (the de novo formation of blood vessels) and angiogenesis (creation of new vessels from existing vessels) together create the backbone of the placental vascular network, which becomes increasingly complex as gestation progresses to term. Many different *ex vivo* imaging modalities, including computed tomography, synchrotron X-ray tomography, confocal, and electron microscopy, and traditional histology and stereology, have been used to study the vascularity of the human placenta in detail. However, many of these techniques often come with trade-offs between resolution, maximum sample size and cost. Light sheet fluorescence microscopy (LSFM) is an attractive method capable of imaging placental 3D morphology at the micro-scale, whilst also enabling imaging of comparatively larger placental explants than other microscopy techniques, allowing capture of more generations of villous and vascular trees. Advances in tissue clearing for optical imaging have led to a multitude of different published LSFM protocols across tissue types. However, optimizing placenta-specific protocols is imperative for high-quality imaging and downstream three-dimensional analysis of this unique tissue. Finally, the placental vascular architecture is notoriously disorganized, heterogenous, and convoluted, making it more challenging to segment and obtain meaningful data than from vasculature in other organ systems. Here, we present a comprehensive pipeline for placental tissue preparation, light sheet fluorescence imaging and image segmentation of first trimester and term human placental villous explants, enabling physiologically relevant quantitative analysis of vascularity at different stages of gestation.

## Teaser

We present a comprehensive pipeline for light sheet fluorescence microscopy of human placental tissue including; tissue preparation, imaging, dataset segmentation, and analysis, with adaptations and considerations for working with first trimester and term samples.

## Introduction

The feto-placental vascular network that forms within the branching villous tissue of the placenta facilitates oxygen and nutrient exchange between the fetus and the mother, which is critical for fetal growth. Placental blood vessel development begins ~15–21 days post conception [1, 2] and as gestation proceeds, this network expands extensively such that by the third trimester the network is dense, extensively branched and convoluted, and able to meet the ever increasing demands of the fetus [3]. The chorionic villi housing this vascular network is stratified. Stem villi (the earliest generations branching from the chorionic plate) have the largest diameter (100–3000 μm) and branch into intermediate villi (80–120 μm), then finally the terminal villi at the end of the tree (~80 μm) [4, 5]. The terminal villi are the main functional unit of the placenta, and are capillary dense with over 50% of the villous containing blood vessels by term [4, 5]. The heterogenous nature of the placental vascular architecture within the villous tissue influences placental hemodynamics, such that the cord insertion point, vascular branching generations, and placental shape all have impacts on vascular resistance, blood flow, shear stress, oxygen diffusion, and exchange capacity [6, 7]. Aberrant placental vascular development is observed in many pregnancy pathologies, including fetal growth restriction, preeclampsia and gestational diabetes [8–10].

A range of *ex vivo* imaging modalities have been used to visualize the three-dimensional (3D) villous and vascular architecture of the placenta across multiple scales, including traditional histology, electron and confocal microscopy, optical coherence tomography, computed tomography, micro-computed tomography (μCT), and synchrotron X-ray computed tomography (sCT) [11]. Each technique offers distinct strengths and limitations, particularly around sample preparation, image segmentation, and throughput, which affect the ability to perform meaningful quantitative analyses of placental structure and support downstream data use [11]. To date, much of the knowledge of placental villous and vascular anatomy has stemmed from histological studies and stereological analyses of two-dimensional (2D) sections [12, 13]. Although stereological design for estimating 3D morphology has become more sophisticated, it remains limited to generalized morphological metrics such as volume, density, length, diameter, and surface area [14–17]. Importantly, 2D approaches cannot consider how morphometrics change across the placental tree, which is critical to infer true structure–function relationships in the feto-placental circulation. Consequently, understanding the true branching complexity of the villous and vascular trees of the placenta, from the macro- to microvascular levels, remains challenging. Light sheet fluorescence microscopy (LSFM) is an emerging modality that enables high-resolution 3D imaging of much larger sample volumes compared with other 3D fluorescence microscopy techniques (e.g. confocal or two-photon microscopy) [18]. As such, it can be used to generate 3D placental imaging datasets at volumes and resolutions similar to those achieved with μCT or sCT, but additionally enable fluorescent labelling of multiple structures or cell types of interest [19].

Adequate tissue clearing of larger samples is critical for achieving high-quality images and resolving internal structures. However, the clearing process has long presented challenges in optical imaging. Biological tissues contain many internal structures (e.g. lipids, elastin, and interstitial fluids) with differing refractive indices (RI). If the tissue is not sufficiently cleared, mismatches in RIs across differing tissue architectures (and often surrounding immersion media) can result in light scatter and suboptimal contrast with depth. Many different protocols and reagents have emerged for optical clearing, including hydrogel-based, organic solvent-based and hydrophilic methods [20–22]; however, these often have tissue-specific performance and identifying an optimal method can be time consuming and expensive. Within these protocols, incubation times and solution volumes also vary widely and are influenced by the density and structure of the tissue of interest, further complicating adaptation to the placental tissue type. Another hurdle in optical imaging is ensuring that the fluorophores’ emission and absorption peaks differ and are compatible with the chosen clearing agent, so as not to dampen or abolish the fluorescent signal [23]. Optical clearing of placental explants for confocal microscopy has been successfully done [24, 25]. However, the optimization of these protocols for application in LSFM enables direct measurement of this 3D morphology in the micro-scale, while also imaging more generations of the villous and vascular trees (not limited to the capillary convolutes) than is possible with confocal microscopy due to the limited light penetration and illumination of the sample from one light source reducing the field of view.

Here, we present an optimized LSFM imaging and analysis pipeline for reproducible, scalable, high-quality 3D placental imaging to quantify placental villous and vascular architecture across gestation. The aim of this work is to enable robust morphological and branching analysis for downstream biological and computational applications.

## Methods

The below optimized LSFM imaging and analysis pipeline is divided into two sections: (1) tissue processing and optical clearing and (2) 3D image processing and analysis. The list of reagents (Table 1), buffers and solutions (Table 2.) and equipment and software (Table 3) required is given below.

**Table 1:** Reagents

Name of Reagent	Company	Catalog number	Comments / Description
30% Hydrogen peroxide	Sigma-Aldrich	H1009-100ML	Bleaching constituent
Alexa Fluor 488 Anti-Cytokeratin 7 antibody	ABCAM	EPR1619Y	Fluorescent staining Host species: Rabbit Clonality: Monoclonal Isotype: IgG
Alexa Fluor 647 Anti-CD31 antibody	ABCAM	EPR17259	Fluorescent staining Host species: Rabbit Clonality: Monoclonal Isotype: IgG
DI H_2_O	N/A	N/A	Diluent
Dimethyl sulfoxide	ThermoFisher	J66650.AE	Buffer constituent
Ethanol	Sigma-Aldrich	E7023-1 L	Ethanol series, sample storage
Glycine	Sigma-Aldrich	G8898	Buffer constituent
Heparin sodium salt	Sigma-Aldrich	H3393	Buffer constituent
HISTO-1	Visikol	H1-30	Optical clearing
HISTO-2	Visikol	H2-100	Optical clearing
Methanol	Sigma-Aldrich	34,860-100ML-R	Bleaching constituent
Normal goat serum	Jackson ImmunoResearch	005-000-121	Blocking constituent
Paraformaldehyde	Sigma-Aldrich	P6148	Tissue fixation
Tween-20	Sigma-Aldrich	P1379	Buffer constituent

**Table 2:** Buffers and Solutions

Buffer / Solution	Final working composition	Diluent
4% PFA	4% PFA	PBS
Antibody Solution	1:300 - Alexa Fluor 488 Anti-cytokeratin 7 antibody 1:300 - Alexa Fluor 647 Anti-CD31 antibody	Blocking buffer
Bleaching Solution	1:4:1 ratio 30% H₂O₂, methanol, DMSO	Methanol, DMSO
Blocking buffer	0.2% Tween-20 / 6% serum / 10% DMSO	PBS
Ethanol series	50% 80% 100%	PBS DI H_2_O
Penetration buffer	0.2% Tween20 / 0.3 M glycine / 20% DMSO	PBS
Phosphate-buffered saline	137 mM NaCl, 1.8 mM KH₂PO4, 2.7 mM KCl, 10 mM Na2HPO4, pH 7.4	DI H_2_O
Washing buffer	**10× Stock:** 0.2% Tween-20 + 10 μg/ml heparin **Working:** 0.02% Tween-20 / 1 μg/mL heparin	stock solution prepared in, and then further diluted 1:10 with PBS

**Table 3:** Equipment and software

Name of equipment	Company	Catalog number	Comments / Description
Polypropylene tubes	Interlab	BCS-500S	Required for compatibility with clearing reagents
Dissection petri dish​	DMT	300412​	For tissue handling and preparation
Dissection pins	DMT	400626​	For tissue handling and preparation
Dissection microscope	Nikon	SMZ800N	Dissection
Fine scissors	Geuder	G-19745	For tissue micro-dissection
Forceps	Dumont	1108-5-PS	For handling tissue samples
Kimwipes	Interlab	110900	For removal of excess liquid from samples
Light sheet fluorescence microscope	PhaseView	Alpha^3^ Facility Edition	Imaging
Petri dishes	ThermoFisher	101VR20	For tissue washing and handling
Scalpel	Swann-Morton	ESS_73252-45_10	For placental dissection
Universal tube	Mediray	C9025SU	For incubation of tissue in solutions
Imaris	Oxford instruments	N/A	Software for 3D visualization & rendering And first trimester image segmentation & filament tracing
Imaris file converter	Oxford instruments	N/A	Conversion of raw files from acquisition to Imaris compatible .ims files
Imaris stitcher	Oxford instruments	N/A	Stitching of tiled datasets to a single dataset for image reconstruction, segmentation and analysis
ITK-SNAP	University of Pennsylvania	N/A	Open-source software for 3D visualization & manual segmentation correction
3D Slicer	Slicer.org	N/A	Open-source software for 3D visualization & segmentation
QtSPIM	PhaseView	N/A	LSFM acquisition software

### Placental collection

The collection and use of all placentae in this research was approved by the Northern X Ethics committee (NTX/12/06/057/AM12). Normal term placentae were collected with informed written consent from Auckland City Hospital following vaginal delivery or caesarean section. Exclusion criteria for term placentae included: multi-parous pregnancies (e.g. twins), placenta praevia, pre-eclampsia, maternal hypertension, auto-immune disease, any class of diabetes, congenital abnormalities, or infections. First trimester placentae were collected with informed written consent following elective surgical termination of pregnancy at Auckland Medical Aid Centre. First trimester placental donors were anonymous to researchers, but were screened by clinic staff so that placentae from pregnancies with any known chromosomal or gross structural abnormalities were excluded.

## Tissue processing and optical clearing

### Tissue preparation

Due to the size and structural composition of placental tissue obtained at different stages of gestation, tissue dissection differs between first trimester and term samples. In both cases, under a stereomicroscope (SMZ800N, Nikon), intact villous trees were dissected to allow imaging of the connected vascular networks throughout each tree.

#### First trimester placentae

Villous tissue was dissected away from the fetal membranes (Figure 1), and a single villous branching tree 1 cm in length, down to the terminal villi, was trimmed to obtain an explant of approximately 1 cm^3^. Explants were then briefly washed in phosphate-buffered saline in 100 mm × 15 mm petri dishes with agitation to remove maternal blood and loosen the villous tissue.

**Figure 1:**
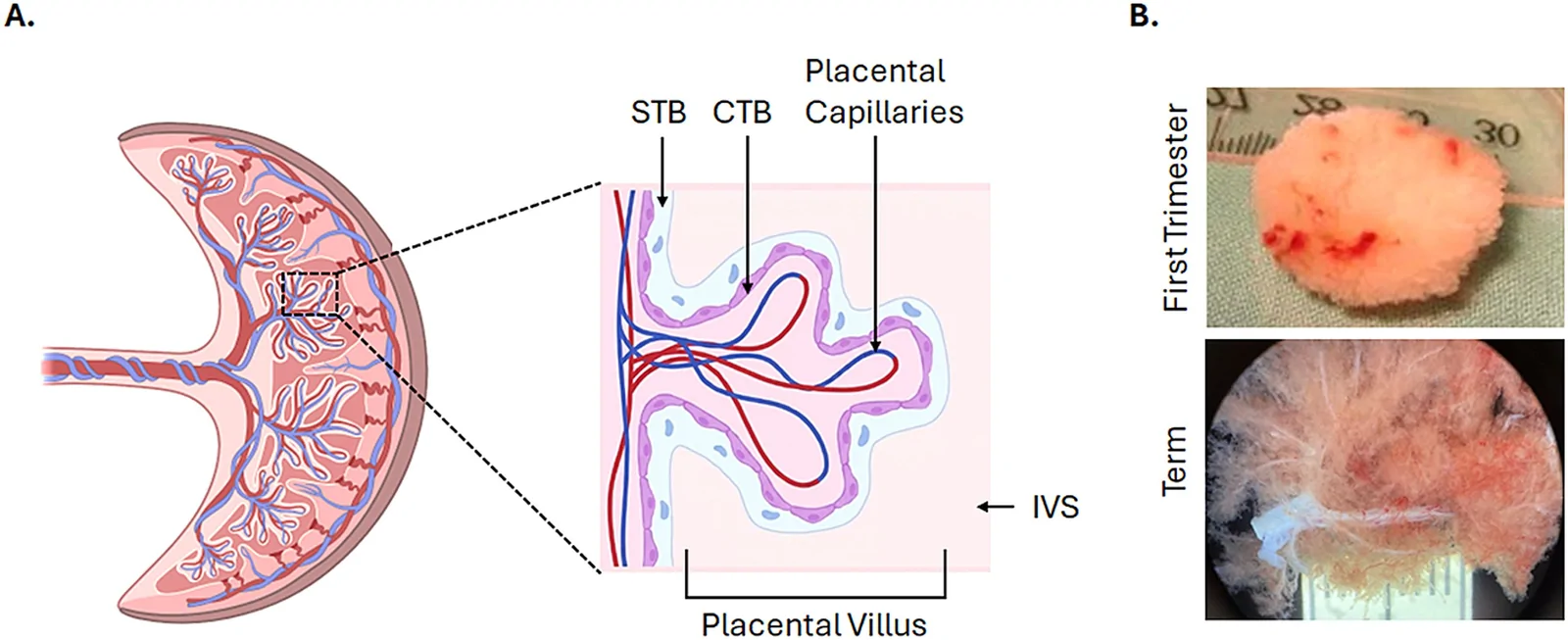
Placental villous explants dissected for light sheet fluorescence microscopy. (A) Placental villi consist of a trophoblast bilayer with an outer syncytiotrophoblast (STB), inner cytotrophoblasts (CTBs), and a mesenchymal core housing feto-placental blood vessels. Villi extend into the intervillous space (IVS), where they are bathed in maternal blood. Created in BioRender. Spring, M. (2026) https://BioRender.com/w5o66y2 (B) First trimester and term tissue isolated for villous explant dissection.

#### Term placentae

Term villous explants were obtained from a cotyledon in the center of the placenta. The decidual layer of the placenta was dissected away leaving 27 cm^3^ explants (3×3×3 cm) (Figure 1), including the chorionic plate. The explants were washed in phosphate-buffered saline (PBS) three times for 10 minutes each. Tissue was then pinned to a high contrast dissection dish and suspended in PBS. Similar to the dissection of first trimester samples, from a stem villus, an intact villous tree of approximately 0.5 cm in length was dissected out to obtain a volume of 0.125 cm^3^ (approximately 0.5×0.5×0.5 cm).

Following dissection of explants, the downstream tissue preparation steps for imaging were the same for both first trimester and term placental explants (Figure 2).

**Figure 2:**
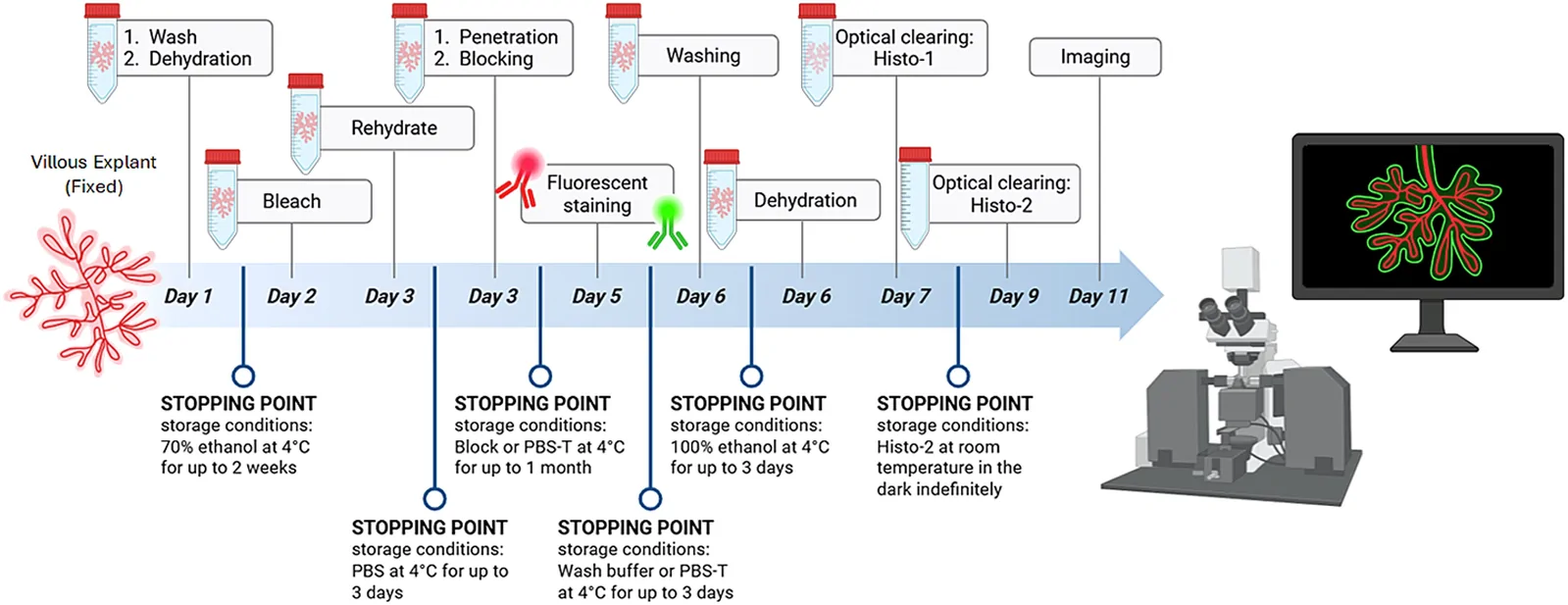
Placental tissue staining and optical clearing timeline for LSFM. Villous explants were dissected from first trimester and term placentae and fixed in 4% PFA. Prior to immunostaining, explants underwent washing, dehydration, bleaching, washing again, penetration, and blocking steps. Samples were then incubated with flurophore-conjugated primary antibodies, then washed, dehydrated, optically cleared using Visikol® HISTO-1 and HISTO-2 and imaged. Created in BioRender. Spring, M. (2026) https://BioRender.com/t2r1xrk

NOTE*: The protocol should commence from the tissue fixation approximately 2 weeks prior to imaging.*

### Tissue fixation

Villous explants were fixed in 4% paraformaldehyde (Sigma-Aldrich, Germany) in PBS overnight at room temperature. The following morning, explants were washed in PBS for 1 hour (2 × 30 minutes).

NOTE: *PBS should not contain calcium, magnesium, or phenol red.*

NOTE: *Explants can be stored in 70% ethanol at room temperature for up to 1 month prior to immunostaining and optical clearing without detrimental effect to tissue structure or staining.*

### Bleaching

This protocol was developed for tissue explants between 0.5 and 1.5 cm^3^. Longer incubation times may be required for larger explants. Villous samples were dehydrated in 6 mL of increasing concentrations of ethanol (50% ethanol in PBS, then 80% ethanol in DI H_2_O, then 100% ethanol) for 1 h at each step using gentle agitation at 4°C. Samples were then transferred to 6 mL of bleaching solution (ice-cold 5% H₂O₂ [from 30% stock], 66.67% methanol, and 16.67% dimethyl sulfoxide (DMSO) or 1:4:1 ratio of 30% H₂O₂/methanol/DMSO) and maintained with gentle agitation at 4°C overnight.

NOTE: *Bleaching of the tissue is required to minimize autofluorescence caused by endogenous pigments such as heme. If autofluorescence is still observed within the vascular network, washing times can be increased to remove any unwanted hemoglobin retained within the vascular network.*

NOTE: *Methanol may inhibit an antibody’s ability to properly bind to their target proteins, particularly at high concentrations. Although methanol in the bleaching buffer was not a notable problem with the antibodies used in this protocol, if loss of signal is apparent consider substituting methanol for ethanol.*

### Immunofluorescent labelling

Samples were rehydrated for staining using a graded ethanol series (6 mL per step; 80% ethanol in DMSO, 80% ethanol in DI H_2_O, 50% ethanol in PBS, then finally 100% PBS), for 1 h at each step using gentle agitation at 4°C. Samples were then incubated in 6 mL of Penetration Buffer (PBS-Tween 20 (PBS-T) / 0.3 M glycine / 20% DMSO) with gentle agitation at 4°C for 1 h. Blocking was performed over 48 h at 4°C with gentle agitation using 6% normal goat serum (NGS) in PBS-T containing 10% DMSO. To label the vascular endothelial cells and outer trophoblast layer of the villi, samples were then incubated in a solution containing two antibodies: 1.67 μg/mL Alexa Fluor 647 anti-CD31 (ab305210; Abcam, UK, endothelial marker) and 1.67 μg/mL Alexa Fluor 488 anti-cytokeratin 7 (ab185048; Abcam, UK, trophoblast marker), diluted in PBS-T, 3% NGS, and 5% DMSO at 4°C with gentle agitation overnight in the dark. Following incubation, samples were washed three times for 30 min each in Washing Buffer (1 μg/mL heparin in PBS-T) with gentle agitation at 4°C in the dark.

### Optical clearing

Optical clearing (Figure 3) was used to increase the transparency of the tissue sufficiently to ensure the LSFM laser penetrated and excited fluorophores within the depth of the explant volume. Samples were first dehydrated in increasing concentrations of ethanol (6 mL per step; 50% ethanol in PBS, 80% ethanol in DI H_2_O, then 100% ethanol) for 1 h at each step using gentle agitation at 4°C. Excess ethanol was removed with Kimwipe laboratory tissues and the tissue was optically cleared using 5 mL of Visikol® HISTO-1 (Sigma-Aldrich, Germany) with gentle agitation at 4°C for 48 h. Clearing was finished in 5 mL of Visikol® HISTO-2 (Sigma-Aldrich, Germany) with gentle agitation at 4°C for minimum of 48 hours. Explants were stored in Visikol® HISTO-2 at room temperature, protected from light until imaging. Longer-term storage of samples for up to 2 months has not been found to compromise or diminish imaging quality.

**Figure 3:**
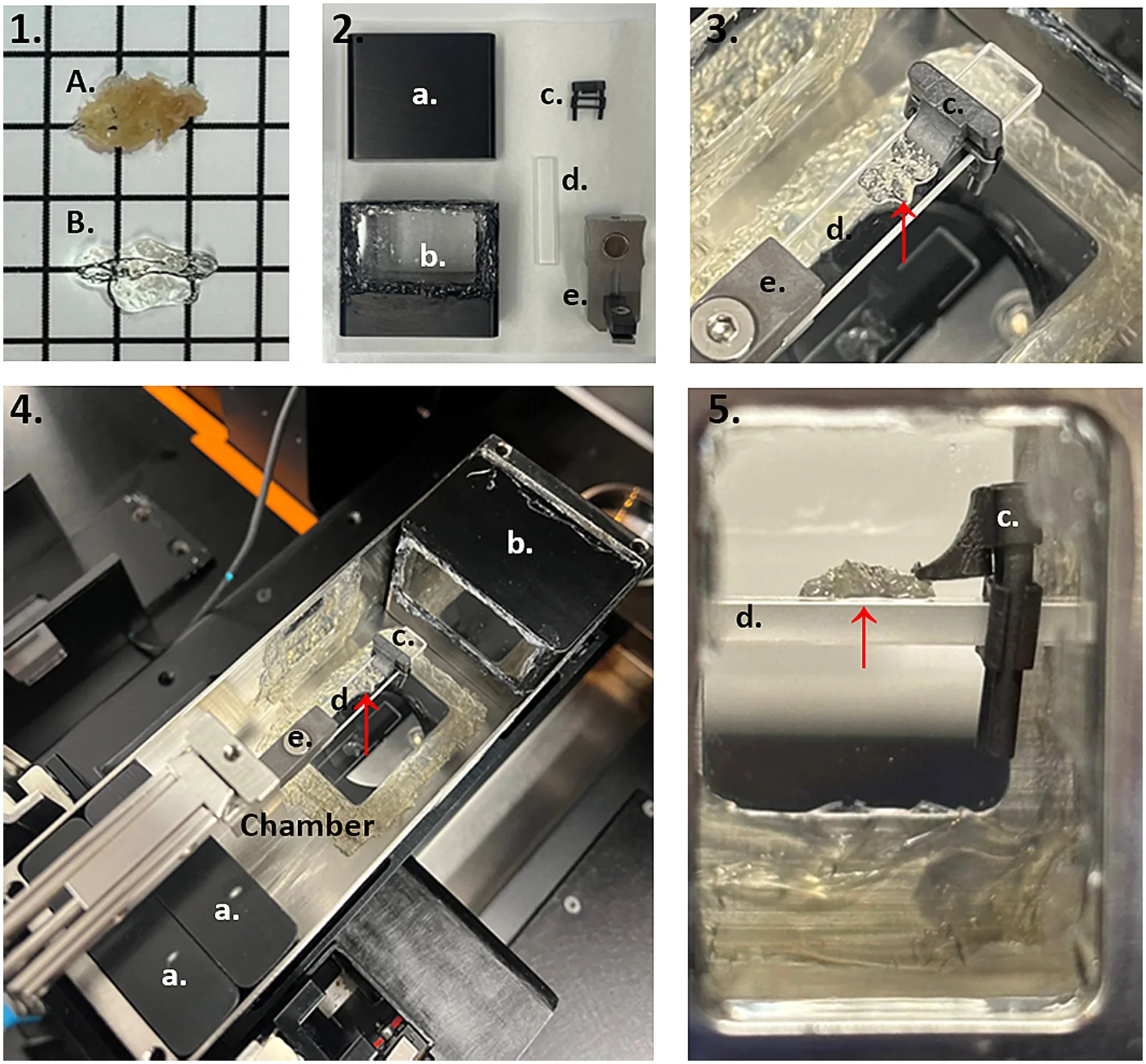
Optical clearing and mounting of villous tissue. (1) First trimester placental explant before (A) and after (B) optical clearing with Visikol® Histo-1 and Histo-2 to increase imaging depth of the laser light sheet within the villous tissue. Grid lines 0.5×0.5 cm. (2) Components required for light sheet imaging (a) = filler bricks, (b) = filler brick with glass viewing window, (c) = Teflon clamp, (d) = glass stage, (e) = holder for glass stage. 3,4,5) A first trimester placental explant mounted on a glass stage within an empty light sheet imaging chamber.

### Light sheet fluorescence microscopy

Optically cleared placental villous explants were imaged using a PhaseView Alpha^3^ Facility Edition light sheet fluorescence microscope (PhaseView, France), controlled by QtSPIM acquisition software (v5.0.5). Samples were gently held on a glass stage with a Teflon clamp by their stalks (the largest villous segment of the tree) to minimize movement during imaging (Figure 3).

NOTE: *It is advisable to dissect larger explants than that intended to be imaged to allow for surplus tissue to be clamped. It is important to minimize tissue movement during acquisition to limit imaging artifacts.*

The imaging chamber was filled with Visikol® HISTO-2 (RI: 1.53) to ensure optical matching with the cleared tissue and reduce light scattering.

NOTE: *Visikol® HISTO-2 can be collected from the chamber following imaging, filtered and reused.*

NOTE: *Custom powder-coated aluminum blocks were fitted into the imaging chamber to reduce the volume of Visikol® HISTO-2 required to submerge the tissue.*

Laser excitation provided by 488 and 638 nm laser lines (108 mW each; Oxxius, France), was used to excite Alexa Fluor 488 and Alexa Fluor 647, conjugated to anti-CK7 and anti-CD31 antibodies, respectively.

Due to the increasing complexity of the vascular network throughout gestation, a higher-magnification objective is required to resolve the architecture at term compared with that in first trimester tissue. For first trimester explants, an XLFLUOR 4×/0.28 NA detection objective (Olympus) in the vertical plane was used to collect emitted fluorescence excited by a laser light sheet of 4 μm thickness produced by two parallel 5×/0.14 NA illumination objectives (Mitutoyo) in the horizontal plane. Resultant images were captured at 1.62×1.63×2.00 μm voxel resolution. For term explants, An XPLN SVMP 10×/0.6 NA detection objective (Olympus) was used in conjunction with 10×/0.28 NA illumination objectives (Mitutoyo) shaping a laser light sheet of 1.5 μm thickness. Resultant images were captured at 0.65×0.65×1 μm voxel resolution. Approximate size of both first and third trimester datasets: 3500×3000×750 pixels.

For both sample types, the emission filter for the 488 nm channel was ET525/50 m and ET690/50 m for the 638 nm channel. Z-stacks of optical sections were acquired sequentially for each fluorescence channel, at optimized intervals of 2 μm (first trimester) or 1 μm (term), representing a step size of half of the light sheet thickness. This provides optimal sampling and sufficient overlap between consecutive Z-planes, providing detailed sampling along the Z-axis.

NOTE: *While smaller Z-step sizes can be used, they increase acquisition time and file size without substantially improving image quality.*

Images were captured using an ORCA-Flash 4.0 V3 camera (Hamamatsu Photonics, Japan) with an exposure time of 70 ms for both channels. Focus sweeping, which moves the thinnest part of the light sheet across a wider field of view to improve image sharpness, was applied to all term tissue samples but not first trimester.

NOTE: *Focus sweeping should be used if live-view inspection confirms improved image clarity, although the increased acquisition time should be considered in decision making. For samples already well focused and evenly illuminated, where improvements in quality are expected to be minimal, focus sweeping is not required.*

X-Y tiling was employed to capture the entire explant when a single field of view was insufficient, typically using 2×2 or 2×1 tile configurations. All imaging was performed in the dark to minimize fluorescence bleaching. Raw multichannel Z-stack images were exported from QtSPIM as .xml files.

## Image processing and analysis

### 3D rendering and image reconstruction

Imaris File Converter (v9.9; Oxford Instruments, UK) was used to convert the raw files into an Imaris-compatible format. Tiled datasets were then stitched using Imaris Stitcher (v10.2.0; Oxford Instruments, UK) to generate a single contiguous volume per explant.

### Image processing

Background subtraction, the use of a Gaussian filter to define the background at each voxel and then performing a baseline subtraction, was applied in Imaris to both 488 nm and 638 nm channels to remove diffuse non-specific staining and background autofluorescence. Filter strengths were selected based on the maximum thickness of strong CK7-positive (30 μm) or CD31-positive (90 μm) staining along the outer trophoblast and endothelial cell layer, respectively (Figure 4).

**Figure 4:**
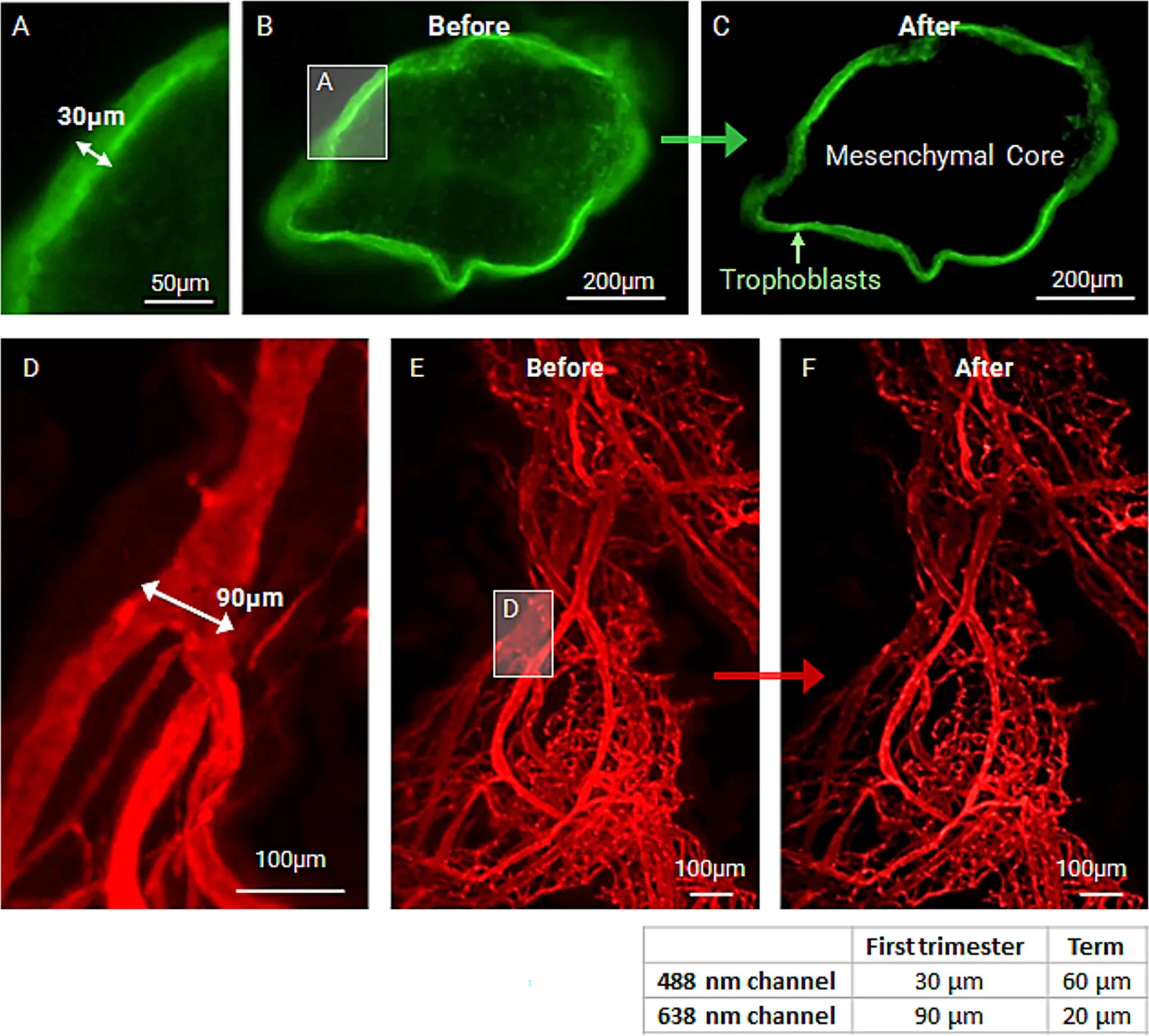
Background subtraction using Gaussian filters. Top row: Background subtraction of the CK7 channel (488 nm) in first trimester tissue. (A) The maximum thickness of strong CK7 staining (~30 μm) along the trophoblast layer defined the Gaussian filter width for subtraction. (B) Villus cross-section before subtraction, showing intense peripheral CK7 staining and weak mesenchymal core signal. (C) After subtraction (filter width = 30 μm), CK7 staining is confined to the trophoblast layer. Bottom row: Background subtraction of the CD31 vasculature channel (638 nm) in first trimester tissue. (A) Maximum vessel diameter (~90 μm) used to define Gaussian filter width for subtraction. (B) Representative 3D rendering of the vasculature prior to and (C) after background subtraction (filter width = 90 μm), showing enhanced contrast. Table: Gaussian filters applied to first trimester and term tissue.

NOTE: *Filter radius selection should be guided by balancing the true thickness of the structure with the risk of classifying it as background, which can cause signal loss and fragmentation of the vascular or villous tree.*

### 3D image segmentation and analysis

Different image-segmentation pipelines were used for first trimester and term datasets due to the increased density, convolution, and structural complexity of term tissue (Figure 5 and Video 1 and 2).

**Figure 5:**
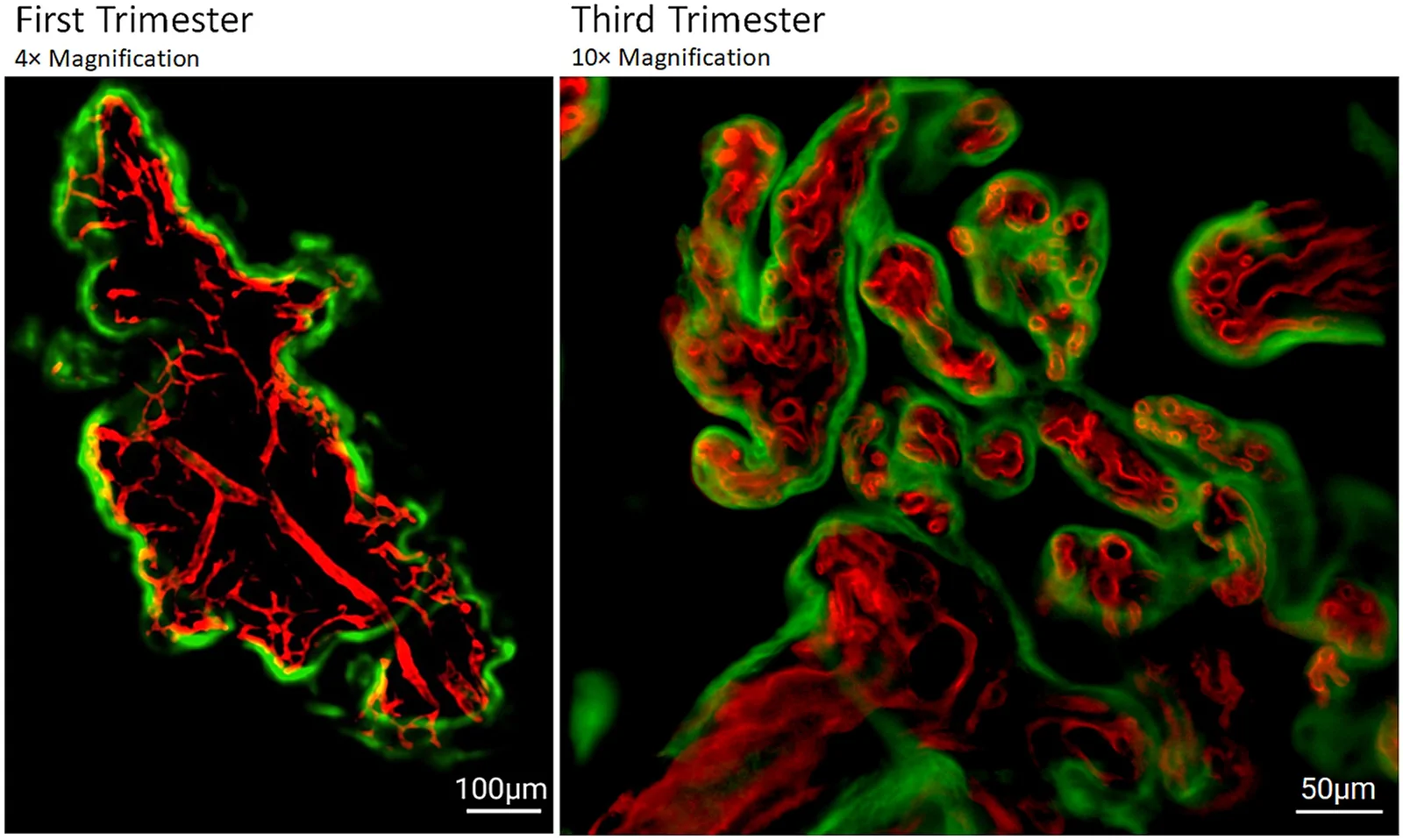
2D sections from 3D light sheet florescence microscopy datasets of first and third trimester placenta. Vascular structure complexity increases across gestation therefore analysis strategies require adaption when moving from first trimester to term placental tissue.

#### First trimester

##### Tissue segmentation

Stitched .ims files were subjected to quantitative image analysis using a pipeline developed in Imaris (v10.2.0; Oxford Instruments, UK). The CD31 channel (638 nm) was segmented using the *Surfaces* function, which applies a machine learning based classification “*Classifier”* to distinguish vascular network from background, enabling quantification of the blood vessel volume. The *Classifier* model was iteratively trained on a region of interest (ROI) within the sample and the *Surfaces* segmentation recomputed until the result visually and quantitatively conformed to the CD31 staining (Figure 6). The ROI was deliberately selected to include areas of the image containing both large and small vessels, ensuring representative training of all vascular structures. A ROI constituting approximately 30% of the imaging dataset (1000×1700 ×600 μm, variable by sample) was sufficient to train the *Classifier*.

**Figure 6:**
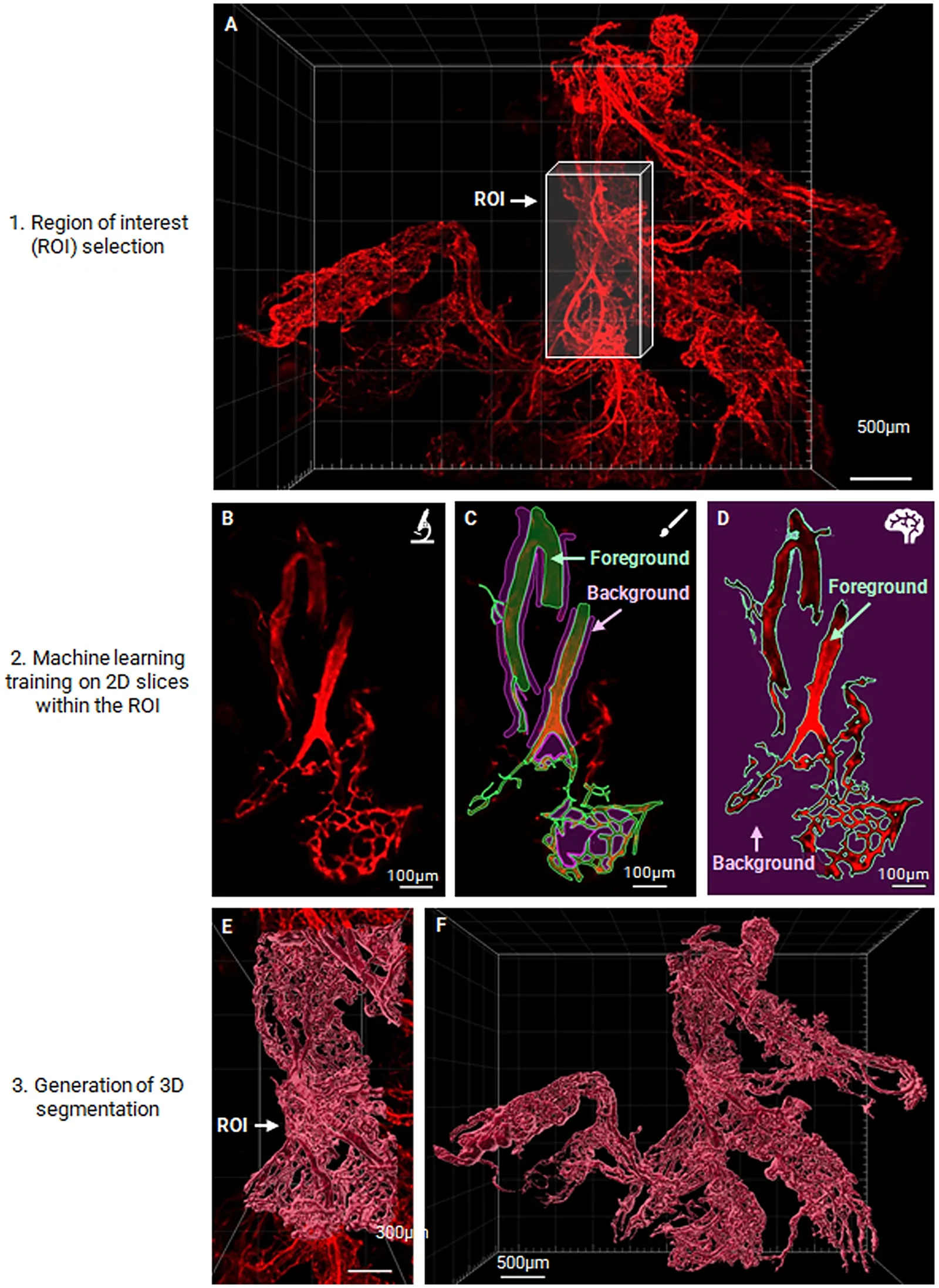
Machine learning-based vasculature segmentation pipeline for first trimester vascular networks using the Surfaces module in Imaris. (A) 3D reconstruction of the CD31-labelled vasculature channel (638 nm) acquired by LSFM. A region of interest (ROI) was selected for training. (B) Representative 2D slice from the ROI. (C) Manual annotation of foreground (vessels) and background using the brush tool. (D) Segmentation prediction generated by Imaris. (E) 3D rendering of vascular segmentation within the ROI, used to assess training performance. (F) Trained model applied to the full 3D reconstruction to generate a vascular segmentation for the entire explant.

The background corrected CK7 channel (488 nm), representing the villous tissue, was segmented using the *Surfaces* function following the same machine learning-based approach used to segment the vascular volume, enabling characterization of villous tissue and calculation of the villous volume. To facilitate segmentation of the full villous volume rather than only the villous surface, background subtraction of the 488 channel was omitted, thereby retaining a low-level autofluorescence signal within the mesenchymal core. Villous tissue and background were manually annotated within regions of interest by navigating through the X-Y plane. Due to heterogeneity between explants, segmentation could not be automated as a batch process, and each image was segmented via an independent process. The vascular volume was normalized to villous volume in order to determine the vascular density (vascular volume/villous volume).

##### Skeletonization and analysis

To generate a graph-based reconstruction of the placental vascular network, enabling quantification of additional vascular parameters including length, diameter, straightness, branching angles and branchpoint density, the segmented vascular geometry generated above was used to perform filament tracing with the *Filaments* module in Imaris.

A binary mask of the segmented vasculature (voxel intensity: 1.00 inside, 0.00 outside) served as the source for filament tracing, providing a high contrast template for accurate seed point placement. Seed points automatically placed within vessels by the software serve to initiate tracing of the vascular network. To accommodate the range of vessel diameters present in placentae, a multipoint detection model was used, whereby seed points of varying sizes are employed. The *AutoPath* algorithm was employed to trace filaments, i.e. linear structures representing vessels, based on local intensity contrast between seed points (Andor Technology, 2022). Using a binary mask of the segmented vasculature, where all voxels within vessels have uniform intensity, greatly enhances this process by guiding filaments along the centre of each vessel and reducing deviations caused by uneven fluorescence or background noise (Figure 7).

**Figure 7:**
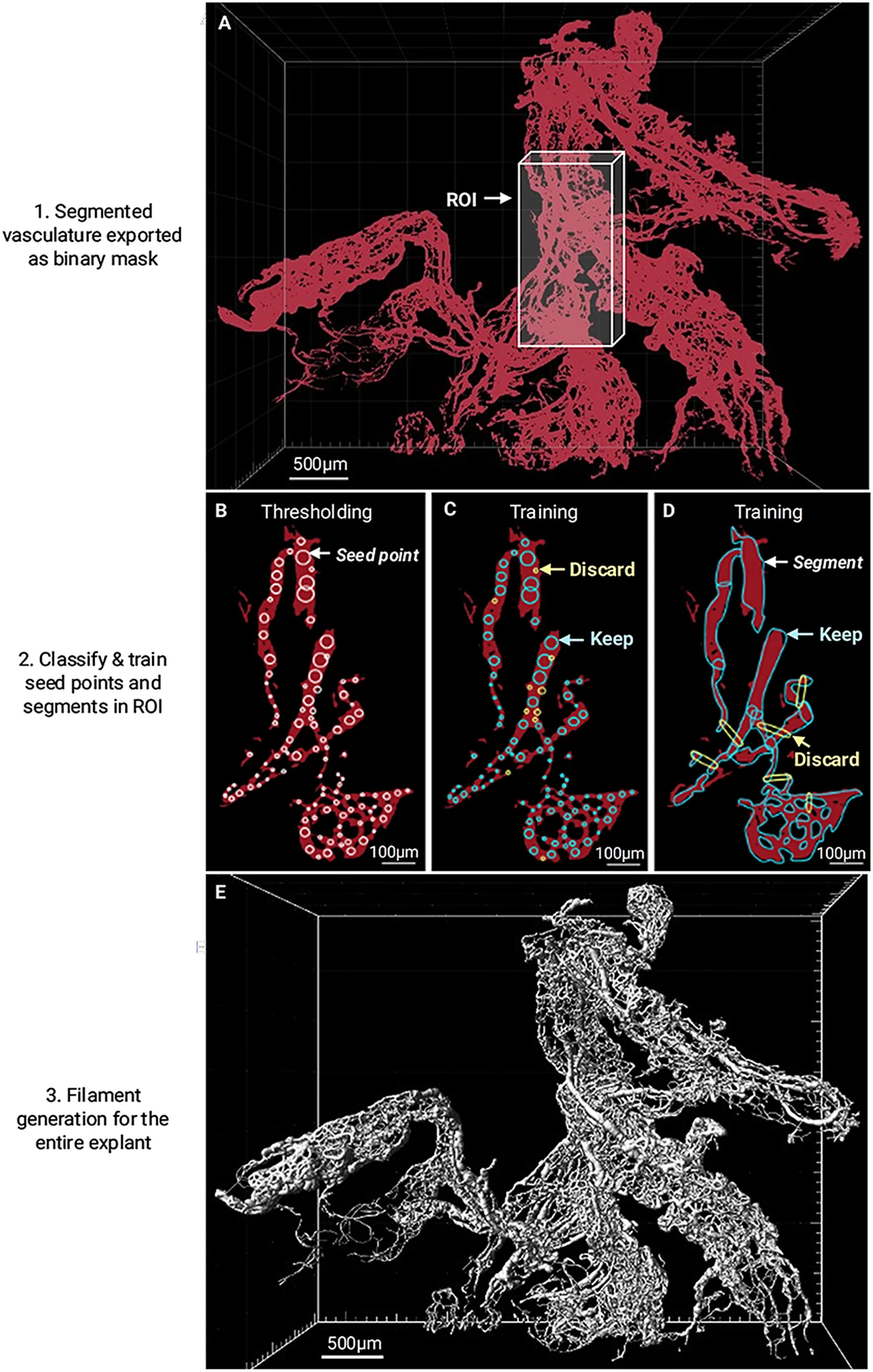
Machine learning-based filament generation pipeline for first trimester vascular networks using the *Filaments* module in Imaris. (A) Segmented vasculature was exported as a binary mask with the region of interest (ROI) for filament tracing training indicated. (B) Representative 2D slice from the ROI showing the optimized seed point density threshold. (C) Seed points classified as “keep” (blue) or “discard” (yellow) for training. (D) Generated segments classified as “keep” (blue) or “discard” (yellow) for training. (E) Filament structure derived after the trained model was applied to the full binary mask of the segmented vasculature.

#### Third trimester

##### Tissue segmentation

During pipeline optimization, automatic segmentation tools within Imaris were deemed unsuitable for term samples. This was primarily because they were unable to preserve a discrete, biologically accurate vascular tree from the stem villus through its downstream branches. While some studies characterize vascular branching without constraining to physiological bifurcating trees, data from LSFM is of sufficient quality that vascular branching can be characterized by more sophisticated methods, including allocation of Strahler ordering to trees and robust quantification of branching properties. Therefore, alternative methods were developed to obtain branching metrics across Strahler orders with semi-automatic and manual approaches employed to preserve the integrity of the branching architecture.

Following background subtraction, term tissue datasets were exported from Imaris into TIF format. The full 3D dataset was mosaicked into eight smaller datasets. Complete image sets were divided by a 2×2×2 grid of images (e.g. mosaic piece size: 1726×1562×735 pixels, variable by sample), and each mosaic piece was segmented individually as the reduced file size made them less computationally demanding to work with. Image processing was conducted using SimpleITK [26] unless otherwise stated. Each mosaic piece was semi-automatically segmented using a combination of 3D-Slicer and ITK-Snap. First, 3D Slicer was used to create a binary mask of the vasculature using a manually selected threshold that reflected the vasculature well. The threshold should be based on biological plausibility, preserving vessel continuity with minimal fragmentation while avoiding over-exaggerating vessel thicknesses. ITK-Snap was then used to manually correct the mask, filling vessel lumens which lacked staining, and separating vessels and capillaries in very close proximity that were incorrectly joined together during thresholding. Manual corrections were done slice by slice in the X-Y plane which led to some thin erroneous connections and inconsistencies along the X-Z and Y-Z planes. To address this, binary opening was applied with a 1-voxel line kernel, in line in the Z axis (Figure 7).

##### Skeletonization and analysis

Following manual corrections, mosaic pieces were reconstructed to create a complete 3D dataset. The segmented image was then skeletonized in 3D using Lee’s algorithm implemented in scipy [27], in which the vessel medial axis was extracted from the mapped binary mask. This vascular skeleton was then converted into a graph of the vascular tree [28] with elements representing tree branches and nodes denoting branching points used to calculate vessel morphometrics; vessel volume, volume fraction, surface area and surface fraction [6, 7, 29] (Figure 8).

**Figure 8:**
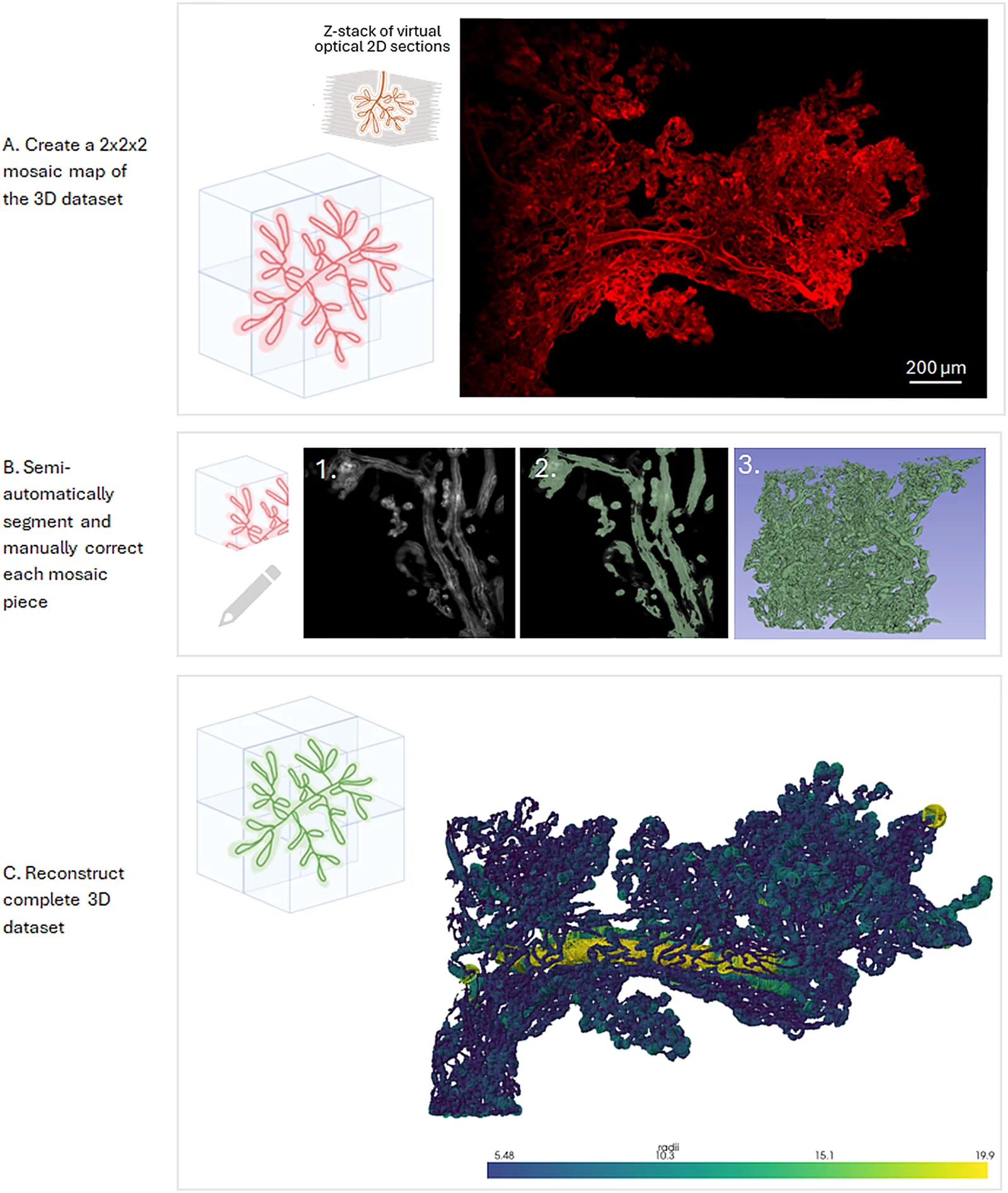
Term semi-automatic vasculature segmentation pipeline. (A) CD31-labelled vasculature channel (638 nm) imaging dataset acquired by LSFM mosaiced into eight smaller datasets for segmentation. (B) Individual cubes (1) were first thresholded in 3D Slicer (2) and (3) to make an initial mask. Hole filling and disentangling joined vessels was manually corrected in ITKsnap. (C) Cubes were reassembled for downstream skeletonization and image analysis.

## Discussion

The methodology presented here integrates refined clearing, immunolabelling, and imaging insights to overcome inherent challenges in preparing placental tissue for optical imaging and introduces two post-processing workflows for handling first trimester and term imaging datasets that differ structurally and require distinct considerations. Over the first trimester vasculogenesis and angiogenesis together create the backbone of the placental vascular network, which becomes increasingly complex as gestation progresses to term. The ability to image capillaries, cells, and molecular markers within the placenta is a strength of fluorescence microscopy, as these structures are harder to distinguish and impossible to identify in other larger scale imaging modalities (X-ray–based approaches). Additionally, LSFM over other imaging modalities (such as confocal or electron microscopy) provides the ability to directly quantify 3D morphology at the microscale while also capturing multiple generations of the villous and vascular trees. Therefore, quantification of these 3D placental images requires custom-built approaches due to the multi-scale, heterogenous nature of the networks. Combining LSFM imaging with dedicated segmentation and analysis pipeline enables direct visualization and quantification of the complex placental anatomy in a physiologically relevant, intact 3D context for downstream comparisons across gestation, or in pathology.

The tissue preparation and imaging protocol presented here helps overcome key limitations of optical imaging of placental tissue, including penetration depth, tissue shrinkage, and fluorescence considerations. Visikol® HISTO clearing reagents were chosen as they were reported to minimize shrinkage and contraction. Although these are solvent-based reagents, it remains possible that some degree of distortion may occur. Corrective adjustments may be considered when interpreting villous and vascular morphometrics [30, 31]. Ethanol-induced tissue shrinkage should also be considered if samples have been stored longer term, although effects are largely confined to the first ~2 weeks of storage, after which tissue dimensions stabilize [32, 33]. Fluorophores in this protocol were specifically chosen for their distinct emission and absorption peaks and compatibility with the Visikol® HISTO clearing reagents, to avoid both dampening or abolishing the fluorescence signal and clear distinction of structures in each channel [23]. Finally, the data presented illustrates the advantages of increased penetration depth and volume imaging that can be achieved with LSFM while maintaining high levels of contrast. As imaging depth increases in optical microscopy, the emitted fluorescence must traverse through more tissue before reaching the detection objective and residual RI inhomogeneities cause scattering and absorption despite clearing. In LSFM placental villous explants are illuminated from each side by two thin laser light sheets, with emitted fluorescence collected by the detection objective perpendicularly along the narrow focal plane. These results in less out-of-focus excitation and photobleaching and reductions in background compared to confocal microscopy (in which illumination and detection both occur through the same objective). Additionally, the speed of acquisition due to the LSFM camera sensitivity and fast readout speed outperforms point-scanning confocal microscopies slower point by point scanning acquisition.

While Imaris is a popular and powerful 3D visualization software for optical imaging, segmentation and analysis software limitations did arise when working with term placental datasets compared to first trimester in which the pipeline was more robust. The term vascular network has larger vessels than first trimester resulting in hollow tubes in the mature network with relatively thin walls and low signal intensity in the lumen. This makes working with Imaris’ surface and filament-based segmentation tools challenging as the software is trained to segment solid, high-contrast structures, which led to vessel walls being partially segmented while the lumens were ignored, resulting in discontinuous or incomplete vessel representations being pronounced in these datasets. In contrast, first trimester tissue walls tend to be thicker and lumens smaller [34] meaning this was less of a limitation in early gestation and first trimester samples were thus more compatible with Imaris’ segmentation assumptions. When working with large imaging datasets, mosaicking the data into smaller file sizes as was done in this work can be highly advantageous if computing power is limited. This method can work well with the range of free open-source programs such as labkit in ImageJ, ITKsnap and 3D Slicer for semi-automatic image segmentation. Following image segmentation, segmented datasets may either be imported into Imaris for filament tracing and branching analysis, consistent with the first trimester workflow, or analysed using open-source 3D branching analysis pipelines implemented in Python [6, 7].

Imaris-based vascular reconstruction and quantification provides a user-friendly, streamlined analytical framework for extracting first-trimester placental vascular metrics, including vessel length, vascular density, branching angle, and branching density. The machine-learning framework significantly reduces analysis time compared with manual segmentation, particularly for villous volume quantification. However, the structural complexity of the placental vasculature precluded batch processing and necessitated more extensive machine-learning training. The Filament Tracer algorithm was originally developed for relatively clean, continuous neuronal networks, whereas early-gestation placental vasculature is dynamically forming through vasculogenesis and branching angiogenesis. Consequently, regions of immature endothelial cords frequently coexist with more mature vascular networks, resulting in filament reconstructions that often comprise partially connected trees reflecting genuine developmental immaturity. Metrics derived from these filament structures must therefore be interpreted within the context of these software assumptions. Nonetheless, the reproducibility of extracted parameters across explants and the broad concordance with values reported by Bierens et al. (2025) support the validity of this analytical pipeline.

For term tissue imaging datasets, the post-processing workflow presented here overcomes challenges that arise from the complexity of the placental architecture, which is greater than that of the first trimester. Working with large, complex 3D datasets is computationally expensive, and whilst there have been advances in computer vision, machine learning, and high-performance compute, there remains a need to combine semi-automatic and manual segmentation in term datasets to correct non-physiological discrepancies and ensure data is analysed accurately. This is especially important when analyzing structures of interest that are challenging to disentangle such as dense vascular networks and terminal capillary loops [11]. Where more crude metrics such as overall volume densities are appropriate to sufficiently answer biological questions, there remains value in automatic segmentation approaches in term tissue to speed up workflows and enable sufficient sample numbers to be analyzed.

Extraction of the 3D term vasculature allows structural and morphological analysis as mentioned above, while identification of the arterial and venous vessels in the future would allow modelling of vessel or network hemodynamics. LSFM is well suited for 3D modelling applications due to its ability to capture larger volumes while also resolving villous tissue structures at multiple length scales. Visualizing both the fetal capillaries and the villous architecture enables exploration of maternal and fetal hemodynamics throughout gestation and in placental complications. This 3D structural data could be used to inform parameters of placenta network models [7, 35], porous media models [36], computational fluid dynamic models [36], or fluid–structure interaction models. Additionally, the ability to image intact vessels at multiple length scales over large fields of view could be used to define representative volume elements (RVEs) that capture the structural heterogeneity and multiscale nature of the branching structures. These RVEs could be integrated with clinical measurements of maternal or fetal hemodynamics (e.g. Doppler ultrasound imaging) to better understand the structure–function relationship of the placenta. Furthermore, coupling the 3D vasculature with the CK7 staining representing the syncytiotrophoblast would allow reaction–diffusion modelling of tissue perfusion in the terminal villous [37], which is critical to understanding the relationship between the villous trees placental function. LSFM additionally allows for staining of multiple proteins of interest beyond just structural markers of the vasculature, which opens up opportunities to obtain spatial information of functional markers within a 3D structure. In this way, the distribution of transporters, signaling molecules or metabolic markers could be explored and linked to computational models of blood flow to better understand the relationship between hemodynamics, 3D architecture, and cellular function in the placenta.

In conclusion, this work presents optimized tissue preparation and optical clearing techniques to improve LSFM imaging of placental tissue, and in doing so has highlighted how LSFM can enhance visualization of the placental vascular network. By quantifying morphometrics such as branching levels, size, angles, tortuosity, and connectivity across multiple generations, one can not only gain a better understanding of differences across gestation or in pathology, but provide physiologically accurate data to employ in multiscale *in silico* approaches that provide insight into the functional consequences of structural vascular aberrations [6, 7, 38].

## Supplementary Material

Supplementary_material_ioag166

## Data Availability

Data not directly available within this article due to Indigenous data sovereignty considerations may be shared on reasonable request to the corresponding author.
